# Tamoxifen Therapy Is Associated With Altered Intestinal P‐Glycoprotein Activity *In Vivo*

**DOI:** 10.1002/jcph.70256

**Published:** 2026-08-12

**Authors:** Álef Machado Gomes Pego, Fernanda de Lima Moreira, Ana Flavia Mendes Batista, Adriana Rocha, Maria Paula Marques Pereira, Eduardo Barbosa Coelho, Jurandyr Moreira de Andrade, Geraldo Duarte, Vera Lucia Lanchote, João Paulo Bianchi Ximenez

**Affiliations:** ^1^ School of Pharmaceutical Sciences of Ribeirão Preto University of São Paulo Ribeirão Preto São Paulo Brazil; ^2^ Faculty of Pharmacy Federal University of Rio de Janeiro Rio de Janeiro Rio de Janeiro Brazil; ^3^ Ribeirão Preto Medical School University of São Paulo Ribeirão Preto São Paulo Brazil

**Keywords:** drug transporters, fexofenadine, p‐glycoprotein, pharmacokinetics, tamoxifen

## Abstract

Preclinical studies suggest that tamoxifen (TAM) may interact with the efflux transporter P‐glycoprotein (P‐gp); however, its effect on intestinal P‐gp activity *in vivo* remains poorly characterized. This study evaluated intestinal P‐gp activity in women receiving tamoxifen therapy using fexofenadine (FEXO) as a probe substrate. Sixteen women receiving tamoxifen (20 mg/day for ≥80 days) and 12 healthy women were enrolled. All participants received a single oral dose of fexofenadine (120 mg), and serial plasma samples were collected over 12 h. Pharmacokinetic parameters were determined by noncompartmental analysis, and plasma fexofenadine concentrations were quantified by LC‐MS/MS. Systemic exposure to fexofenadine was lower in women receiving tamoxifen than in healthy women. Geometric mean (95% confidence interval [CI]) AUC_0‐12h_ was 646.55 ng.h/mL (523.50–798.52) in the tamoxifen group and 994.77 ng.h/mL (832.21–1189.07) in healthy women, whereas AUC0‐∞ was 715.40 ng.h/mL (588.92–869.05) and 1123.66 ng.h/mL (919.98–1372.43), respectively (*P* < .05). Apparent oral clearance (CL/F) was higher in women receiving tamoxifen (167.74 vs 106.79 L/h; *P* < .05), whereas elimination half‐life was unchanged between groups. The observed pharmacokinetic profile is consistent with reduced oral bioavailability and suggests modulation of intestinal transporter‐mediated drug disposition during tamoxifen therapy. These findings may have implications for the disposition of concomitantly administered orally administered P‐gp substrates and warrant further investigation in dedicated clinical pharmacology studies.

## Introduction

Breast cancer (BC) remains the most frequently diagnosed malignancy among women worldwide.^1^, ^2^ Advances in early detection and systemic treatment have markedly improved survival outcomes, with 5‐year relative survival rates exceeding 90% in several populations.^3^, ^4^ Given the long‐term use of adjuvant therapies in this setting, understanding potential pharmacological interactions has become increasingly relevant.

Tamoxifen (TAM) constitutes a cornerstone of adjuvant endocrine therapy for estrogen receptor‐positive (ER‐positive) breast cancer. In women, it is commonly prescribed for at least 5 years, either as monotherapy or in combination with ovarian function suppression in higher‐risk cases.^5^, ^6^ As a selective estrogen receptor modulator (SERM), tamoxifen exerts antagonistic effects in breast tissue while acting as a partial agonist in bone and endometrial tissues. Five years of adjuvant therapy are associated with an approximate 31% reduction in recurrence and mortality risk.^7^ Tamoxifen is extensively metabolized by CYP2D6 and CYP3A enzymes, and CPIC guidelines recognize CYP2D6 activity as an important determinant of endoxifen formation and tamoxifen bioactivation.^8^ Given its widespread and prolonged use, potential effects of tamoxifen on additional pathways involved in drug disposition, including membrane transporters, are also of clinical interest.^9^

ATP‐binding cassette (ABC) transporters contribute substantially to interindividual variability in systemic drug exposure.^10^, ^11^ P‐glycoprotein (P‐gp), encoded by the *ABCB1* gene, is an ATP‐dependent efflux transporter expressed in several barrier tissues, including the apical membrane of enterocytes in the small intestine. At this site, P‐gp limits oral drug absorption by transporting substrates back into the intestinal lumen, thereby reducing systemic bioavailability.^12^, ^13^

Preclinical evidence suggests that tamoxifen may modulate P‐gp transporter function. *In vitro* studies have demonstrated that tamoxifen stimulates P‐gp ATPase activity, indicating a potential functional interaction with this efflux transporter.^9^, ^14^ These findings raise the possibility that tamoxifen therapy may alter intestinal transporter‐mediated drug absorption *in vivo*. If such modulation results in increased intestinal P‐gp activity, reduced systemic exposure to orally administered P‐gp substrates would be expected. However, despite these mechanistic observations, clinical evidence evaluating the effect of tamoxifen therapy on intestinal P‐gp activity in humans remains limited. Therefore, the *in vivo* impact of tamoxifen on intestinal P‐gp activity has not been clearly established.

Fexofenadine (FEXO) is recommended by regulatory agencies, including the US Food and Drug Administration (FDA), as a probe substrate for the assessment of intestinal P‐gp activity.^15^, ^16^ It is a non‐sedating H1 receptor antagonist characterized by low oral bioavailability, minimal metabolism, and predominant excretion as unchanged drug.^17^, ^18^ Its pharmacokinetics are also predictable, with linear exposure observed after single or repeated administration at doses up to 120 mg twice daily, supporting its suitability as a probe substrate in clinical pharmacology studies.^19^ Studies in *Mdr1* knockout animal models demonstrate increased systemic exposure to fexofenadine following oral administration, supporting the role of intestinal P‐gp in limiting its absorption.^20^ Although fexofenadine may also interact with uptake transporters such as OATPs,^21^ it remains a widely accepted and clinically safe probe for intestinal P‐gp phenotyping.^22^

Given the widespread use of tamoxifen and the potential role of transporter‐mediated mechanisms in drug exposure variability, it is clinically relevant to determine whether tamoxifen therapy alters intestinal P‐gp activity *in vivo*. Therefore, the present study aimed to evaluate *in vivo* intestinal P‐gp activity in women receiving tamoxifen therapy compared with healthy women, using fexofenadine as a probe substrate.

## Methods

### Patients and Data Collection

**Figure 1:**
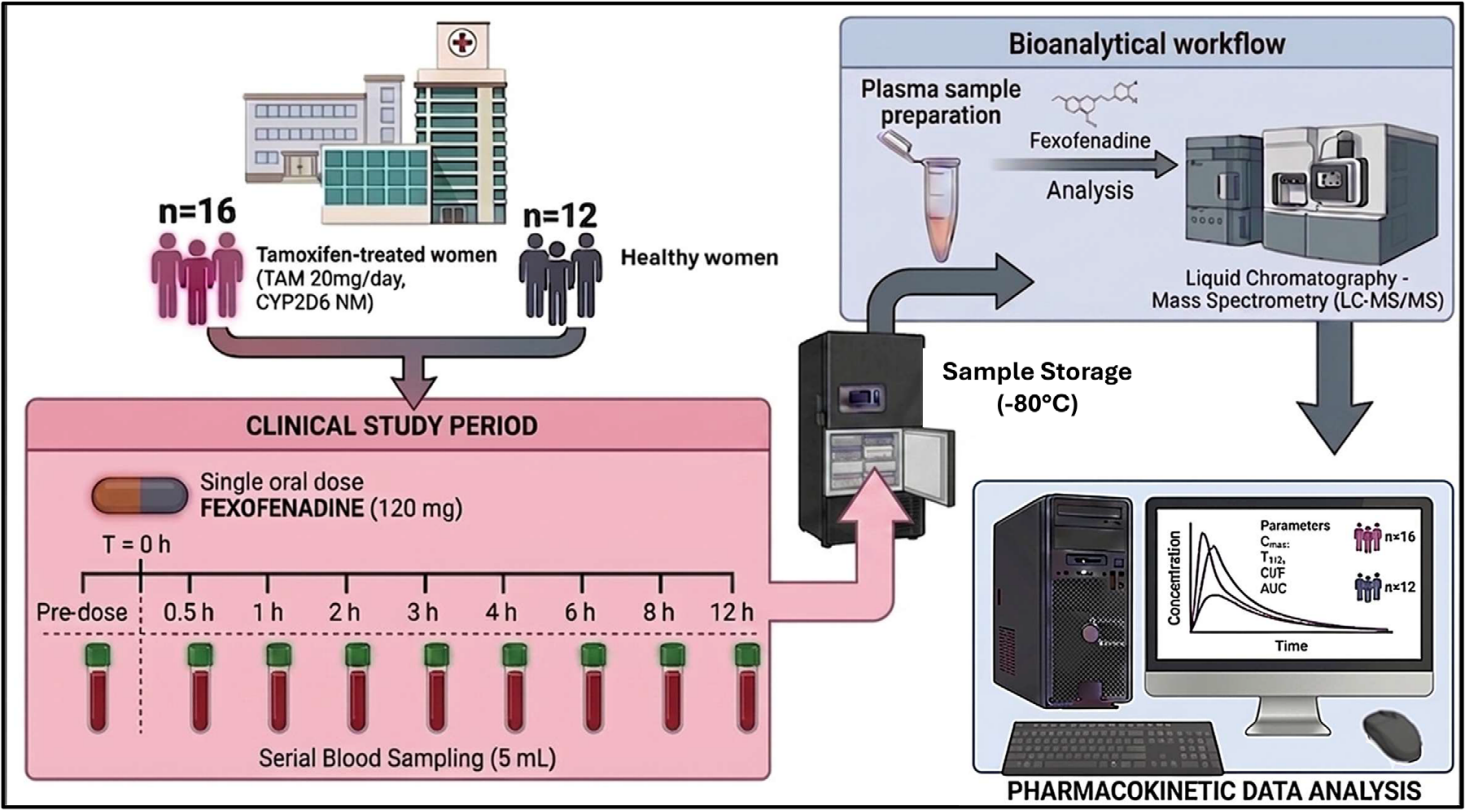
Schematic representation of the study design and analytical workflow. Tamoxifen‐treated women (20 mg/day, CYP2D6 normal metabolizers [NM], n = 16) and healthy women (n = 12) received a single oral dose of fexofenadine (120 mg). Serial blood samples were collected from pre‐dose to 12 h post‐dose. Plasma fexofenadine concentrations were quantified by liquid chromatography‐tandem mass spectrometry (LC‐MS/MS). Pharmacokinetic parameters, including Cmax, AUC, CL/F, and t1/2, were subsequently derived from plasma concentration‐time data.

This study was designed as an open‐label, parallel‐group comparative clinical pharmacology study based on a secondary pooled analysis of two previously conducted clinical studies using an identical fexofenadine probe protocol (Figure 1). The pooled dataset comprised the tamoxifen‐treated cohort described by Ximenez et al^23^ and the healthy control cohort reported by Moreira et al.^24^ Both source studies were conducted at the same research center and used the same fexofenadine dose, blood sampling schedule, sample processing procedures, and LC‐MS/MS bioanalytical method, enabling direct pharmacokinetic comparison between groups. The studies were approved by the ethics committees of the School of Pharmaceutical Sciences of Ribeirão Preto and the University Hospital of the Ribeirão Preto Medical School, both from the University of São Paulo, Ribeirão Preto, SP, Brazil (record numbers: 35539714.7.0000.5403 and 67037617.3.0000.5403). All participants provided written informed consent prior to enrollment.

Participants were divided into two groups according to clinical status: women receiving tamoxifen therapy and healthy women. The tamoxifen‐treated group included premenopausal women with histologically confirmed ER‐positive breast cancer who had been receiving adjuvant tamoxifen therapy at a dose of 20 mg/day for at least 80 days. Premenopausal status was determined based on menstrual history, ovarian ultrasound features, and age. The healthy women group consisted of women without diagnosed comorbidities and not receiving medications known to affect fexofenadine pharmacokinetics or intestinal P‐gp activity. Participants in both groups were excluded if they had used medications known to induce or inhibit P‐gp within 1 month prior to study initiation.

Each participant received a single oral dose of fexofenadine (120 mg), administered as a probe for intestinal P‐gp activity. Serial blood samples (5 mL) were collected into heparinized tubes at pre‐dose and at 0.5, 1, 2, 3, 4, 6, 8, and 12 h post‐dose and stored at −80°C until analysis.

All participants in the tamoxifen‐treated group were classified as CYP2D6 normal metabolizers (NMs) according to CPIC activity score guidelines, based on prior genotyping performed in this cohort as part of a pharmacogenetic study of tamoxifen therapy.^8^, ^25^

### Sample Size Calculation

Sample size estimation was performed using PS Power and Sample Size Calculation software (version 3.1.2). The study was powered for a two‐sided comparison of independent means, assuming a Type I error rate of 5% and 80% power. A 35% difference in fexofenadine systemic exposure between women receiving tamoxifen as adjuvant therapy and healthy women was considered clinically relevant. Based on previously published pharmacokinetic data in healthy volunteers, mean (SD), AUC 496.67 ng.h/mL (±131),^26^ and assuming equal variances between groups, a minimum of 10 participants per group was required to detect an absolute difference of ±173,83 ng.h/mL in AUC of fexofenadine between groups.

### Analysis of Fexofenadine in Plasma

Plasma samples (50 µL) were spiked with diphenhydramine (10 ng/mL) as the internal standard (IS) and 150 µL of acetonitrile for protein precipitation. After centrifugation, fexofenadine was separated on an Ascentis Express C18 (Merck, Darmstadt, Germany) using a mobile phase composed of water and acetonitrile (60:40, v/v) containing 0.1% formic acid. Quantification was performed on a Micromass Quattro micro triple quadrupole mass spectrometer LC‐MS/MS system (Waters, Milford, USA) operating in the positive electrospray ionization mode, following a previously described method.^24^ The assay showed no matrix effect and was linear over the concentration range of 1–1000 ng/mL. Precision and accuracy were within acceptable analytical limits according to validation guidelines, with coefficients of variation and relative standard errors below 15%.

### Pharmacokinetic Analyses

Fexofenadine pharmacokinetic parameters were derived using noncompartmental analysis implemented in Phoenix WinNonlin, version 8.5.4 (Certara USA, Inc., Princeton, NJ, USA). Maximum observed plasma concentration (C) and time to C_max_ (T_max_) were obtained directly from the individual concentration‐time profiles. The area under the plasma concentration‐time curve from time zero to 12 h (AUC_0‐12h_) was calculated using the linear trapezoidal method. AUC_0‐∞_ was calculated as AUC_0‐12h_ plus the residual area estimated as C_last_/k_el_, where C_last_ represents the last measurable plasma concentration and k_el_ the terminal elimination rate constant. Apparent total clearance (CL/F) was calculated as Dose/AUC_0‐∞_. The elimination half‐life (t_1/2_) was derived as ln(2)/k_el_.^27^

### Genotyping

Genotyping of CYP2D6 polymorphisms in tamoxifen‐treated participants was previously performed in the same cohort as part of a pharmacogenetic investigation of tamoxifen therapy.^28^ In brief, genomic DNA was extracted from peripheral blood, and CYP2D6 variants were determined using validated real‐time PCR‐based allelic discrimination assays, including assessment of gene deletion and copy number variation. CYP2D6 diplotypes were inferred using the HaploStats package in R, version 4.5.2 (R Foundation for Statistical Computing, Vienna, Austria), and star‐allele designations were assigned according to the Pharmacogene Variation Consortium (PharmVar) database. CYP2D6 metabolic phenotypes were determined using the activity score system following CPIC genotype‐to‐phenotype translation guidelines.^8^ A detailed description of the genotyping procedures and analytical methods has been reported by Mendes et al.^28^

### Statistical Analyses

Fexofenadine pharmacokinetic parameters (C_max_, AUC_0‐12h_, AUC_0‐∞_, and CL/F) were evaluated for log‐normal distribution using the Shapiro‐Wilk test. Comparisons of pharmacokinetic parameters between women receiving tamoxifen treatment and healthy women were performed using the unpaired Student's t‐test when data followed a log‐normal distribution and exhibited homogeneous variances. Elimination half‐life (t_1/2_) was compared between groups using the Wilcoxon rank‐sum test. Evidence of a statistical difference was defined as a *P* value < .05. Geometric mean ratios and their corresponding 90% confidence intervals (90% CI) were calculated for the pharmacokinetic parameters. When the 90% CI of the geometric mean ratio for a given parameter was entirely contained within the bioequivalence range of 0.80–1.25, the parameter was considered clinically equivalent between groups.^29^ All statistical analyses were performed using R software, version 4.5.2 (R Foundation for Statistical Computing, Vienna, Austria) using the stats package.

## Results

Sixteen women receiving tamoxifen therapy and 12 healthy women were included in the analysis. Women in the tamoxifen‐treated group were older than healthy controls (48 vs 28 years), whereas body mass index was comparable between groups (28.36 ± 4.27 vs 26.03 ± 2.68 kg/m^2^). Baseline demographic, biochemical, and hematological characteristics are summarized in Table S1.

**Figure 2:**
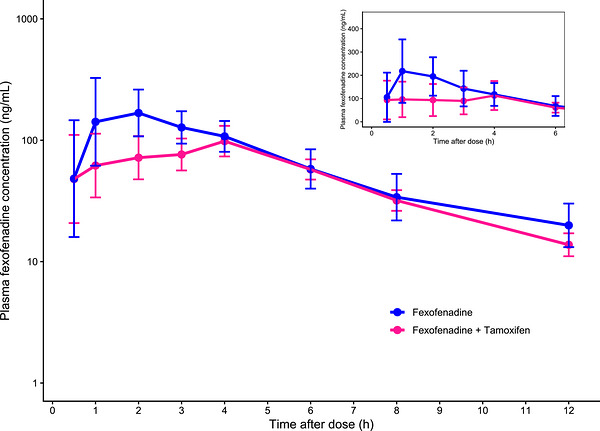
Plasma concentration–time profiles of fexofenadine following a single 120‐mg oral dose in the fexofenadine + tamoxifen group (tamoxifen‐treated women, n = 16) and the fexofenadine‐only group (healthy women, n = 12). Data in the main panel are shown on a logarithmic scale and are presented as geometric mean with 95% confidence interval. The inset panel illustrates the early absorption phase on a linear scale using arithmetic mean ± standard deviation.

The geometric mean plasma concentration‐time profiles of fexofenadine following a single 120‐mg oral dose are presented in Figure 2 and demonstrate consistently lower systemic exposure in women receiving tamoxifen therapy.

**Figure 3:**
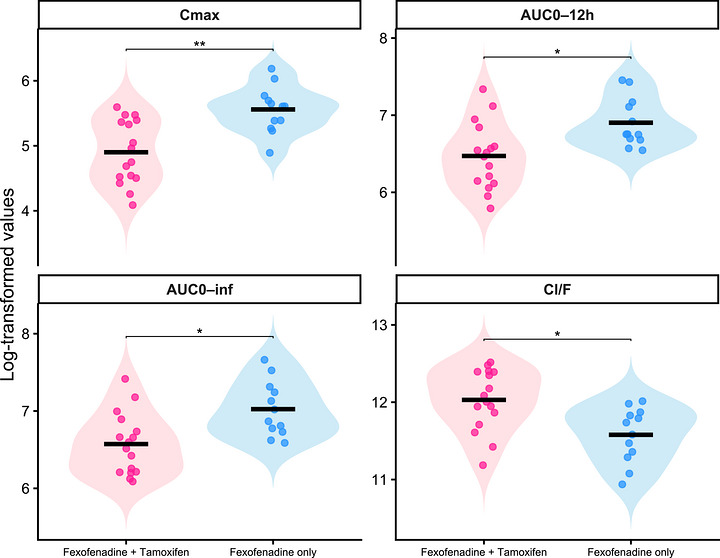
Violin plots showing the distribution of the pharmacokinetic parameters C_max_, AUC0‐12h, AUC0‐∞, and Cl/F of fexofenadine in women receiving tamoxifen treatment (pink) and healthy women (blue). Individual points represent observed values, and the horizontal black line indicates the median. AUC, area under the plasma concentration‐time curve; C_max_, maximum observed plasma concentration; Cl/F, apparent total clearance. *P < .05; **P < .001.

Pharmacokinetic parameters are summarized in Table 1. Peak exposure (C_max_) and total systemic exposure (AUC_0‐12h_ and AUC_0‐∞_) were reduced in the tamoxifen‐treated group compared with healthy women. Geometric mean C_max_ values were 134.35 and 259.58 ng/mL, respectively, whereas AUC_0‐12h_ values were 646.55 and 994.77 ng.h/mL (*P* = .00515), corresponding to an approximate 35% reduction in systemic exposure. Similarly, CL/F was higher in the tamoxifen‐treated group, whereas elimination half‐life (t_1/2_) did not differ significantly between groups. Individual distributions of C_max_, AUC, and CL/F are shown in Figure 3.

**Table 1:** Pharmacokinetic Parameters of Fexofenadine After a Single 120‐mg Oral Dose in Women Receiving Tamoxifen Therapy (FEXO + TAM) and Healthy Women (FEXO)

Parameter	Fexofenadine + Tamoxifen, GM (CI 95%), (n = 16)	Fexofenadine, GM (CI 95%), (n = 12)	GMR, CI 90% (FEXO + TAM/ FEXO)	*P* Value
C_max_ (ng/mL)	134.35 (105.64‐170.87)	259.58 (212.3‐317.37)	0.52 (0.39‐0.69)	.00034
AUC_0‐12h_ (ng.h/mL)	646.55 (523.50‐798.52)	994.77 (832.21‐1189.07)	0.65 (0.51‐0.84)	.00515
AUC_0‐∞_ (ng.h/mL)	715.40 (588.92‐869.05)	1123.66 (919.98‐1372.43)	0.64 (0.50‐0.82)	.00396
Cl/F (L/h)	167.74 (138.08‐203.76)	106.79 (87.44‐130.44)	1.57 (1.23‐2.01)	.00396
t_1/2_ (h)	2.82^*^ (2.57‐3.16)	3.26^*^(2.66‐4.09)	0.93 (0.75‐1.15)	.20530

AUC_0‐12h_, area under the plasma concentration‐time curve from time zero to 12 h; AUC_0‐∞_, area under the plasma concentration‐time curve from time zero to infinity; CI, confidence interval; Cl/F, apparent oral clearance; C_max_, maximum observed plasma concentration; FEXO, fexofenadine; GM, geometric mean; GMR, geometric mean ratio; t_1/2_, the elimination half‐life; TAM, tamoxifen.

Between‐group comparisons were performed using unpaired t‐tests on log‐transformed data.

* t1/2 is presented as median (interquartile range [IQR])

**Figure 4:**
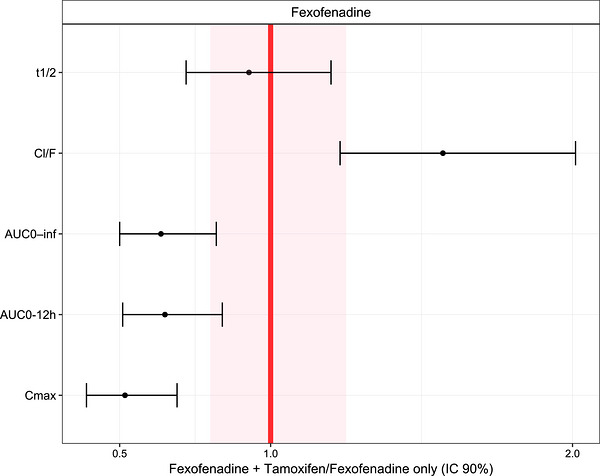
Bioequivalence graphs of the pharmacokinetic parameters of fexofenadine observed in women treated with tamoxifen (n = 16) and healthy women (n = 12). The shaded area represents the bioequivalence range (0.80–1.25). C_max_, maximum plasma concentration; AUC, area under the plasma concentration‐time curve; Cl/F, apparent total clearance. Data are presented as the ratio of geometric means (90% CI).

Geometric mean ratios (GMR: tamoxifen‐treated/healthy) were 0.52 (90% CI 0.39–0.69) for C_max_, 0.65 (90% CI 0.51–0.84) for AUC_0‐12h_, and 0.64 (90% CI 0.50–0.82) for AUC_0‐∞_, confirming lower systemic exposure in women receiving tamoxifen therapy. Consistent with this reduced exposure, CL/F was increased (GMR 1.57, 90% CI 1.23–2.01). For C_max_, AUC, and CL/F, the 90% confidence intervals were outside the conventional bioequivalence range (0.80–1.25), indicating that these parameters did not meet conventional bioequivalence criteria between groups. Elimination half‐life was comparable between groups (GMR 0.93, 90% CI 0.75–1.15; Table 1 and Figure 4).

## Discussion

The present study evaluated intestinal P‐gp activity *in vivo* using fexofenadine as a probe substrate in women receiving tamoxifen therapy (20 mg/day). Compared with healthy women, tamoxifen‐treated participants showed lower systemic exposure to fexofenadine, reflected by reduced AUC_0‐12h_ and AUC_0‐∞_, increased CL/F, and unchanged elimination half‐life. This pharmacokinetic profile is consistent with reduced oral bioavailability and altered intestinal transporter‐mediated drug disposition.

Tamoxifen is extensively biotransformed by cytochrome P450 enzymes, particularly CYP3A4/5 and CYP2D6, generating the active metabolites 4‐hydroxytamoxifen and endoxifen, which contribute substantially to its clinical activity.^9^, ^30^ In parallel with its metabolic pathways, experimental studies have shown that tamoxifen and its metabolites may interact with P‐gp (*ABCB1*).^31^, ^32^ *In vitro* data indicate stimulation of P‐gp ATPase activity within a concentration range suggestive of a direct functional interaction.^9^, ^14^, ^33^ However, most of the available literature has been derived from preclinical systems, and data on the effect of tamoxifen therapy on intestinal P‐gp activity in humans remain limited. In this context, the present study extends the literature by providing *in vivo* clinical data consistent with the modulation of intestinal P‐gp activity during tamoxifen treatment.

Fexofenadine was chosen as a probe to measure P‐gp activity because it has been widely used in transporter phenotyping studies and has an established safety profile.^34^, ^35^, ^36^, ^37^ Its systemic exposure increases in the presence of P‐gp inhibition and decreases with induction, which supports its use as a functional probe of intestinal P‐gp activity.^38^ However, fexofenadine is not a selective intestinal P‐gp probe, as it is also transported by uptake transporters including OATP2B1, OATP1B1, and OATP1B3.^22^, ^39^ For this reason, the present findings are best interpreted as evidence of altered transporter‐mediated disposition, with a profile that is compatible with increased intestinal efflux, rather than as a direct or isolated measure of intestinal P‐gp expression or function.

A coherent pharmacokinetic pattern emerged in women receiving tamoxifen treatment, characterized by lower AUC, higher apparent oral clearance, and unchanged elimination half‐life. Both AUC_0‐12h_ and AUC_0‐∞_ were lower in women receiving tamoxifen therapy, indicating lower systemic exposure after oral administration. For an orally administered substrate, this finding is compatible with increased intestinal efflux and reduced fraction absorbed.^16^, ^40^ The reciprocal increase in CL/F is also consistent with this interpretation. Because CL/F is defined as Dose/AUC_0‐∞_, an increase in apparent oral clearance in this setting is more readily explained by reduced bioavailability than by a change in true systemic clearance. The absence of a difference in elimination half‐life further supports a predominantly pre‐systemic mechanism. Taken together, the combination of lower AUC, higher CL/F, and preserved t_1/2_ suggests that the observed difference is driven mainly by altered absorption‐related processes.

This interpretation is also consistent with previous clinical observations using fexofenadine. Rifampicin, a recognized inducer of intestinal P‐gp, has been reported to reduce fexofenadine exposure without altering elimination half‐life.^41^ Although direct mechanistic comparisons should be made cautiously due to differences in study design and populations, the pharmacokinetic profile observed during tamoxifen therapy is directionally consistent with increased intestinal efflux activity.^42^

An additional aspect of the present study is that all women treated with tamoxifen had previously been classified as CYP2D6 NMs.^28^ Since CYP2D6 is a major determinant of tamoxifen bioactivation,^30^ this reduces an important source of interindividual variability related to tamoxifen disposition within the treated group. Therefore, variability related to CYP2D6‐mediated tamoxifen bioactivation was minimized within the treated group.^25^

Some limitations should be considered when interpreting these findings. Although the study was adequately powered for the primary comparison, the sample size remains modest and limits a more detailed assessment of potential covariates. In addition, *ABCB1* genotyping was not performed, and functional polymorphisms may contribute to interindividual variability in fexofenadine pharmacokinetics.^43^ Potential drug‐disease interactions related to breast cancer itself, as well as unmeasured physiological differences between groups, cannot be completely excluded. In addition, potential gene‐disease interactions, whereby disease‐related physiological changes may interact with underlying genetic variability in transporter expression or function, cannot be excluded. Although participants using known P‐gp modulators were excluded, the contribution of unrecognized concomitant exposures or residual drug‐drug interactions cannot be entirely ruled out. Furthermore, because fexofenadine is also transported by OATP transporters,^22^, ^39^ the present data do not allow exclusive attribution of the observed effects to P‐gp modulation alone.

Although the present study was not designed to evaluate clinical outcomes or exposure‐response relationships, the observed reduction in fexofenadine exposure suggests that tamoxifen therapy may alter the oral disposition of coadministered intestinal P‐gp substrates. For drugs whose efficacy or safety depends on maintaining specific systemic exposure ranges, altered intestinal transporter activity could contribute to clinically meaningful pharmacokinetic variability, particularly for orally administered P‐gp substrates with narrow therapeutic windows or steep exposure‐response relationships.^44^, ^45^, ^46^ If a similar effect occurs with other orally administered P‐gp substrates, reduced systemic exposure could potentially lead to subtherapeutic drug concentrations and diminished therapeutic efficacy. Despite the study limitations, the overall pharmacokinetic profile observed during tamoxifen therapy‐reduced systemic exposure, increased apparent oral clearance, and unchanged elimination half‐life‐remains consistent with altered intestinal efflux transporter‐mediated disposition in vivo. Further prospective clinical pharmacology studies are warranted to clarify the underlying mechanisms and determine whether these transporter‐mediated alterations may justify dose adjustment or therapeutic monitoring in specific clinical settings.

## Conclusion

In conclusion, tamoxifen therapy was associated with reduced systemic exposure to fexofenadine and increased apparent oral clearance, without affecting elimination half‐life. This pharmacokinetic profile is consistent with altered intestinal efflux transporter‐mediated disposition and reduced oral bioavailability *in vivo*. Considering the widespread use of tamoxifen in women receiving long‐term endocrine therapy, these findings may have clinical implications for the disposition of concomitantly administered orally administered transporter substrates. Further clinical pharmacology studies are warranted to clarify the underlying mechanisms and clinical relevance of this interaction.

## Author Contributions

Á.M.G.P., A.F.M.B., V.L.L., and J.P.B.X. wrote the manuscript. Á.M.G.P., F.L.M., E.B.C., J.M.A., G.D., V.L.L., and J.P.B.X. designed the research. Á.M.G.P., F.L.M., M.P.M.P., A.R., F.L.M., and J.P.B.X. performed the research. Á.M.G.P. and J.P.B.X. analyzed the data.

## Conflicts of Interest

The authors have no relevant conflicts of interest to disclose.

## Funding

The authors would like to thank the São Paulo Research Foundation (FAPESP, numbers: 2014/16360‐9, 2018/05616‐3, and 2024/05744‐2) and the Brazilian National Council for Scientific and Technological Development (CNPq) for funding support.

## Supporting information




**Supplementary File 1**: jcph70256‐sup‐0001‐SuppMat.docx

**Supplementary File 1**: jcph70256‐sup‐0002‐ICMJE.pdf

## Data Availability

Original data are available from the corresponding author upon request.
