# Supplementary material for: Tamoxifen Therapy Is Associated With Altered Intestinal P‐Glycoprotein Activity In Vivo
Source: J Clin Pharmacol. 2026 Aug 12;66(8):e70256. doi: 10.1002/jcph.70256 (PMC13464512; doi:10.1002/jcph.70256)
Supplement: Supplementary file 2 [file JCPH-66-0-s002.docx]

**Supplementary material**

Additional data supporting the findings of this study are provided in the Supplementary Material. Supplementary Table S1 summarizes the baseline demographic, anthropometric, biochemical, and hematological characteristics of the study participants.

**Supplementary Table S1**. Baseline demographic, anthropometric, biochemical, and hematological characteristics of women receiving tamoxifen treatment and healthy women. Data are presented as mean ± standard deviation (SD) or median (interquartile range, IQR), as appropriate. Creatinine clearance was estimated using the Cockcroft-Gault equation.

|  | **Women receiving tamoxifen treatment**  n=16 | | **Healthy women**  n=12 | |
| --- | --- | --- | --- | --- |
| ID | Mean | SD | Mean | SD |
| Weight (kg) | 72.83 | 12.83 | 70.34 | 12.14 |
| Height (m) | 1.60 | 0.06 | 1.64 | 0.09 |
| Age (years) | 48.00* | (42 - 49.25) | 28.00* | (24 - 31.25) |
| Body mass index (kgm^2^) | 28.36 | 4.27 | 26.03 | 2.68 |
| Urea (mg/dL) | 28.69 | 8.48 | 27.06 | 4.66 |
| Serum Creatinine (mg/dL) | 0.77 | 0.14 | 0.75 | 0.12 |
| Total Billirubin (mg/dL) | 0.34 | 0.14 | 0.51 | 0.21 |
| Alanine aminotransferase (U/L) | 15.64 | 6.19 | 19.47 | 8.31 |
| Aspartate aminotransferase (U/L) | 17.92 | 5.22 | 20.13 | 11.30 |
| Blood glucose (mg/dL) | 94.44 | 15.33 | 77.77 | 6.31 |
| Gama glutamyl transferase (U/L) | 34.50 | 12.23 | 16.36 | 6.46 |
| Alpha-I acid glicoprotein (mg/dL) | 79.53 | 23.89 | 72.85 | 12.71 |
| Total proteins (g/dL) | 6.54 | 0.42 | 6.67 | 0.62 |
| Hemoglobin (g/dL) | 11.97 | 1.24 | 13.28 | 0.93 |
| Hematocrite (%) | 35.50 | 3.61 | 40.18 | 2.40 |
| Plaquetae (10^3^/Ul) | 224.88 | 42.63 | 271.27 | 55.43 |
| High densite lipoprotein (mg/dL) | 49.39 | 9.66 | 61.23 | 10.92 |
| Trigliceride (mg/dL) | 142.88 | 90.71 | 100.93 | 35.76 |
| Total cholesterol (mg/dL) | 188.50 | 32.07 | 176.78 | 39.35 |
| Low densite lipoprotein (mg/dL) | 115.38 | 30.08 | 99.21 | 36.45 |
| Clearance creatinine (mL/min) | 107.82 | 22.02 | 127.61 | 23.60 |
| Anti-HIV Elisa | NR |  | NR |  |

*data are presented in median (Interquartile interval)
